# Walking on the Number Line: A Playful and Embodied Group Intervention for People with Acquired Number Deficits (Acalculia)

**DOI:** 10.3390/brainsci16090940

**Published:** 2026-09-02

**Authors:** Yael Benn, Georgios Kountouriotis, Verena Christin-Pavel, Maryam Hussain, Tam Dibley, Mark Jayes, Berzan Cetinkaya

**Affiliations:** 1 School of Psychology, Manchester Metropolitan University, 53 Bonsall Street, Manchester M15 6GX, UK; g.kountouriotis@mmu.ac.uk (G.K.); verena.pavel01@gmail.com (V.C.-P.); 2 School of Health Sciences, University of Manchester, Oxford Road, Manchester M13 9PL, UK; maryamhussain45564@gmail.com (M.H.); berzan.cetinkaya@medeniyet.edu.tr (B.C.); 3 School of Education, Manchester Metropolitan University, 53 Bonsall Street, Manchester M15 6GX, UK; tam.dibley@gmail.com; 4 Department of Health Professions, Manchester Metropolitan University, 53 Bonsall Street, Manchester M15 6GX, UK; m.jayes@mmu.ac.uk; 5 School of Health and Related Research, University of Sheffield, Sheffield S10 2TN, UK; 6 Faculty of Health Sciences, Istanbul Medeniyet University, Atalar Mah. Şehit Hakan Kurban Cad. No: 44, 34862 Istanbul, Turkey

**Keywords:** acalculia, stroke, brain-injury, aphasia, rehabilitation, embodied cognition, playfulness, enriched social environment, far-transfer

## Abstract

**Highlights:**

**What are the main findings?**

- Group-intervention for acalculia can be effective and acceptable to users.
- Playful and embodied activities are acceptable and effective in supporting the rehabilitation of stroke survivors.

**What are the implications of the main findings?**

- Support for stroke survivors with acalculia that incorporate sensory-motor tasks into numerical activities may support the development of a ‘number sense’ rather than address specific numerical deficits.
- Future work should systematically examine the effectiveness of group support across multiple domains (wellbeing, cognitive, physiological and functional).

**Abstract:**

Numbers-skills are affected in as many as 65% of cases following a stroke or a brain-injury, a condition termed ‘acalculia’. Post-stroke numerical skills interventions have to-date focused on using intense repetitions (‘drill’), in one-to-one settings. However, while ‘drill’ is needed, there is increasing evidence that learning (and re-learning) of numerical skills may be enhanced by additional techniques, informed by current educational and cognitive neuroscience theories. We report on a mixed-methods feasibility study examining the acceptability and impact of the first reported group intervention for acalculia, that is based on the principles of embodied learning, playfulness, and enriched social environment. Four brain-injury survivors took part in six-weekly 45-min group sessions involving games with numbers, accompanied with congruent movements. Following a 4-week break, *n* = 3 took part in additional three sessions. Performance on number skills (theoretical and functional) was collected before the intervention (T0), after six weeks (T1) and after further three weeks (T2) using validated batteries. Qualitative data were collected 3-months post intervention using semi-structured interviews with two patients. Results show improvements on all measures at both T1 and T2, including far-transfer to untrained problems. Qualitative findings emphasised the importance of group-settings, and the impact of playful learning on cognition, engagement, learning, confidence and wellbeing. We conclude that playful group therapy integrating modern educational theories is feasible and can be effective for improving numerical skills and speculate on the possible neural mechanisms that may facilitate these findings. Future work should evaluate the impact of combining multi-sensory, movement and cognitive rehabilitation in improving patients’ linguistic, physiological, and wellbeing outcomes.

## 1. Introduction

Numerical skills are essential for a healthy and prosperous life. Numerical information is common in healthcare (e.g., nutrition information, blood-sugar readings, probability of risk), in financial management and in everyday life (e.g., understanding temperature, calculating times and distances, using addresses, phone numbers, dates and times). Still, despite the centrality of numerical skills to everyday life, individuals with poor numerical skills, including those resulting from a stroke or brain-injury, have little or no access to support for their numerical skills, with negative impacts on their life [1].

The evidence on the prevalence of numerical deficit after a stroke (acalculia) is inconclusive. Studies suggest that it affects between ~30% and 65% of stroke survivors [2,3]. In comparison, the relatively more well-known condition of aphasia, an acquired language disorder, affects a third of stroke survivors [4].

Despite the negative outcomes of low numeracy and the high prevalence of acalculia, there has been little evidence on interventions for improving numeracy skills following brain-injury. A recent systematic review [5] identified only 16 studies published in English that describe interventions for acalculia, with a total of *n* = 31 participants. Again, contextualising the scarcity of evidence in comparison to interventions for other post-stroke impairments, such as aphasia, a Cochrane review of speech and language therapy for aphasia reported the results of 57 Randomised Control Trials (RCTs) involving *n* = 3002 participants [6].

All published studies for acalculia focus on the rehabilitation of very specific skills. These include multiplication [7,8,9,10,11,12,13,14,15,16], fractions [17], mixed arithmetical operations [18], and transcoding (transfer of numbers from one modality to another, e.g., from oral number words to written digits) [19,20,21,22]. Importantly, in all the studies the interventions were delivered individually (i.e., no group interventions), and most used interventions tailored to the individuals’ deficits, making them difficult to transfer to other cases. In addition, the majority of the interventions used a ‘drill’ strategy, with only a few (e.g., [11,17]) using other strategies (e.g., strategic learning of rules or colouring shapes of fractions respectively). None of the published studies utilised games.

In sum, while the two earliest publications [13,20] used ‘drill’ as the then-current and culturally dominant educational concept for learning math, subsequent work tended to apply adaptations to these original studies, rather than advance in-line with developing educational theories. For example, even recent publications (e.g., [22]) report the outcome of an intervention that is based on these early approaches, rather than test new interventions that are informed by advances in educational theories. As such, the current work seeks to re-introduce and emphasise the importance of current educational thinking in rehabilitation programmes.

Mathematics education, and particularly concepts around informal education, have been transformed in recent years. The following Sections will outline three relevant theoretical concepts in education (embodied-learning, games and playfulness, and Enriched Social Environment for learning), to introduce the thinking behind the design of the current programme.

### 1.1. Embodied Cognition and Learning

The theory of embodied cognition [23] suggests that learning and cognition emerge through the interaction of an organism with an environment, and as a result of integration of sensory-motor activity into a model that is then encoded in memory. Evidence shows that in a classroom situation, students recall information better when embodied-learning techniques are used, particularly when taught with gestures that correspond (are congruent) with the ideas being taught [24].

Numbers are particularly closely linked to the body; children learn to count using their fingers, and studies report motor cortex brain activation in response to adults observing numbers [25]. Several studies have shown that congruent actions can enhance performance on numerical tasks in adults; for example, walking or orienteering ‘forward’ or ‘right’ enhances addition, while walking ‘backwards’ or orienting ‘left’ (as in on a number line) helps subtractions [26,27]. As such, it is believed that math acquisition occurs through active engagement with the environment [28], and that embodied-learning should be used more widely for math education in schools [24].

The developmental literature supports the link between movement and numerical skills. There is a significant relationship between math achievement and the development of Fine Motor Skills (FMS) and Gross Motor Skills (GMS) such that balancing, skipping and star jumps are significantly correlated with school math performance [29] and non-symbolic magnitude processing [30]. The evidence goes beyond observational studies. For example, whole-body movement interventions in children improved performance on spatial positioning of numbers [31,32] and improved accuracy in solving additions and subtractions [27]. Overall, both GMS and FMS predict mathematical achievement in young children [33,34].

The link between embodied-learning and cognition largely focuses on learning in children, but re-learning in adulthood remains largely unexplored. Only a small number of studies have examined the effectiveness of embodied-learning beyond primary education (see for example, with K-12 education: [35]), and we found no evidence of interventions for numerical skills following a stroke or brain-injury that are based on these principles [5].

Within the wider rehabilitation literature, the importance of combining movement (but not necessarily congruent activity, as required by the principles of embodied-learning theory) and cognition has been recognised. A recent systematic review [36] reported that combined and simultaneous physical activity and cognitive interventions are superior for motor rehabilitation outcomes compared to non-combined interventions, and a recent meta-analysis [37] suggests that combined cognitive and physical therapy is superior to cognitive therapy alone. However, numerical skills were not included in the studies measuring cognitive outcomes.

### 1.2. Games and Playfulness for Learning

The importance of games in learning is well-established. A meta-analysis of 30 studies involving games for learning (*n* = 3202 participants) reported a significant positive medium effect when learning with, compared to without, games [38].

Mathematics learning is no exception and can be greatly enhanced by the use of games within schools. Jagušt et al. [39] compared the use of competitive, collaborative, and adaptive tablet-based games for mathematic learning among second- and third-grade students. Results revealed that compared to a non-gaming condition, games contributed to improved math performance in all game conditions. Math learning through games has also been shown to be effective among those with special educational needs [40].

Several reviews have identified the positive impact of digital games on post-stroke cognitive [41,42,43] and physical [44] rehabilitation. However, only one unpublished work has focused on rehabilitation of mathematical skills among stroke-survivors using a digital game [45] and only a few games designed for rehabilitation combine physical activity with cognitive rehabilitation (e.g., [46,47]). Furthermore, these interventions do not typically capitalise on a range of educational theories (e.g., they rarely employ embodied principles).

While games are effective, they often represent a ‘fun’ way to reinforce a ‘drill’ strategy. However, an important component of gaming is ‘playfulness’. While the exact definition of playfulness varies [48], Gadamer [49] suggested that play has its own spirit (e.g., rules), no purpose and its outcome is unknown, hence it involves risk. Playfulness incorporates within it fun-seeking, uninhibitedness, and spontaneity [50], and it has been shown to reduce stress and increase wellbeing [51,52,53], as shown since the introduction of play-therapy by Anna Freud and Virginia Axline [54]. Playfulness can also encourage creativity and seeking out complex and novel challenges within one’s limitations, hence enhancing learning [55]. Mathematical play may be defined as playing with possibilities and constraints as offered by materials, tools, negotiation with others, bending rules, and inhabiting imaginative as-if worlds [56,57]. The intervention described here explores some of these concepts.

### 1.3. Enriched Social Environment

Despite the positive social and wellbeing impact of group work on brain-injury survivors [58], and the benefits of the use of group-settings for cognitive rehabilitation of skills, such as memory [59] and language [60], none of the interventions designed to enhance mathematical cognition to date have been designed as a group intervention [5].

Enriched Social Environment (ESE) is a term used to describe an environment where participants can explore and interact with each other in a multi-stimuli setting that encourages participation in rich social, physical, and cognitive activities simultaneously. An example may be an environment that presents a challenging task to be completed using novel solutions that are developed with others in a team. The importance of such environments for rehabilitation has been widely studied in animals [61]. In humans, rich environment intervention was found effective in Parkinson’s disease patients [62] and was more effective than traditional post-stroke intervention in stroke survivors [63]. However, more evidence across different cognitive domains is needed.

Therefore, the current study seeks to examine the feasibility, impact and acceptability (based on the Theoretical Framework of Acceptability; TFA, [64]) of a group-intervention for acalculia. In particular, the study seeks to examine whether the principles of embodied learning, games and playfulness, and an enriched environment can be effectively used to support and improve numerical skills among brain-injury survivors. To this end, we employ both quantitative evaluation of changes in numerical skills, and a qualitative analysis of interviews with a subset of the programme’s participants.

## 2. Materials and Methods

### 2.1. Participants

Participants were recruited through advertising in local stroke survivors support groups. Four stroke-survivors participants took part in the study, but one participant, who had gone on their summer holiday after the first six weeks, did not participate in the final three sessions of the programme (or the follow up tests). While this sample size is small, it was considered sufficient for a feasibility study, particularly as participants varied in their age (mean = 61.5 years, *SD* = 15.76, range: 46–77 years) gender, aphasia severity, professional role pre-stroke and time post-onset (mean = 29.5 months, *SD* = 21.69, range: 18–62 months) as seen in Table 1 below.

To take part, participants had to be adults over the age of 18, who previously had a stroke or another form of brain-injury that affected their ability to process numerical information. Participants’ language skills were diagnosed using the Western Aphasia Battery (WAB; [65]; see full WAB scores in Appendix B, Table A2). Participants were included regardless of language or physical disability, but they had to be able to provide informed consent. Acalculia sub-type was not diagnosed, given there are currently no assessments to reliably do this.

### 2.2. Procedures

All participants took part in six 45-min weekly group sessions involving activities and games with numbers, accompanied with congruent movements where possible. All sessions were pre-designed, but material for some sessions was slightly adjusted for participants levels (see Table A1, sessions 6 and 7). Following these six sessions, the group was given a 4-week break, during which P4 had gone on his summer break, and so only the remaining three participants continued for three further group sessions of 45 min each. All sessions were delivered by the first author, and supported by the 3rd and 4th authors, and on two occasions, a student-volunteer (between 0 and 3 supporters in each session). During the 4-weeks break, the research team had reviewed the method of delivery to examine if any changes or adaptation were needed, and some of the sessions were adjusted to involve even more creative and participatory materials. It was observed that through increased awareness of their own challenges, and self-comparison with the group, participants started to identify personal goals for development and have expressed the wish for specific practice. As this was perceived by the research team to be a good marker of proactive learning and engagement, and due to the lack of validated software that could be used for home-practice, for the final three sessions, participants were offered the opportunity to add 45 min at the end of the group session to practice specific numerical skills in a 1-to-1 setting. All three participants took on this opportunity, each choosing to practice different skills. P1 chose to practice word-based problems, P2 focused on basic counting and additions, and P3 chose to focus on number naming. These sessions were entirely desk-based practice, without playful, embodiment, or social delivery.

Performance on number skills (theoretical and functional) was collected by the last author (BC), who did not attend the sessions, and was blind to the methods and theoretical concepts behind the work. Testing took place before the intervention (T0), after the first six weeks (T1) and after further three weeks (T2) during 1–2 online sessions (depending on the participant’s fatigue and progress). This was done using two established and validated scales. Qualitative data were collected by the first author (YB) 3-months post intervention using semi-structured interviews with P1 and P2. The interview schedule was developed by YB and MJ (who did not attend the sessions and was blind to the theoretical underpinning/content of the intervention). Data analysis was conducted by YB, and MJ contributed to the analytic process through peer scrutiny and discussions.

### 2.3. Testing of Numerical Skills

Numerical skills were assessed using two types of validated assessments. The Functional Numeracy Assessment (FNA, [66]) is designed as a functional test of numerical skills for people with language difficulties. The task includes ‘everyday’ problems, such as identifying which of several products represent ‘better value’ according to price per unit. While the test is short, specifically designed for participants with aphasia, and it has been validated with English speakers, it was only a pilot study, and is accommodated with limited normed data (from both controls and patients with aphasia). When combined with the observation that its use to date is limited, a second test was included.

The EC301 [67] is a theoretical number-skills test. It requires the participants to perform tasks, such as counting (forward, backward, in steps) and simple calculation. Despite being long (31 items, of which we omitted items 23 and 24, which involved placing numbers on analogic scale, as they could not be completed online, making a total of 29 items and a maximum score of 281), it has been extensively validated across different European countries, though not with English speakers, and some of the questions used in the test may be influenced by aphasic symptoms. We therefore consider the combination of the two tests a solid platform for examining changes in numerical skills as a result of the intervention.

### 2.4. Semi-Structured Qualitative Interviews

This aspect of the work followed closely the procedure described at Benn et al. [1]. Interviews with two of the four participants took place online and were recorded on MS Teams. As the interviews were conducted by the first author, who also delivered the intervention, participants were familiar with the interviewer, and so no extra introductions were needed. Interviews started with general questions about the effect of the stroke in general, moved on to focus on difficulties the participants experienced with numbers and any support they received for it prior to the current intervention, and only then did the questions focus on the participants’ experience with the intervention, by saying “I would like to talk to you a little about your experiences with the intervention for numbers you took part in”. Examples of questions were: ‘How did you feel when you were offered help with numbers?’ ‘What were you expecting to do during the intervention?’ ‘I would now like to ask you a little about the content of the intervention. What activities did you find most helpful/difficult/fun/unhelpful?’ ‘What did you think about working as part of a ‘group’? what did you like/not like about it?’. Lastly, participants were asked about life since the intervention: ‘Have you noticed any difference in the way you engage with numbers in your life? (if so, can you describe it?).

The questions were used flexibly to stimulate discussion, and points were probed and followed up as they were raised. As both patients were able to communicate well online, no prompts were required to support communication, but hand gestures were regularly used and recorded during the transcription process.

The familiarity of the interviewer with the participants and the intervention resulted in some way in richer data, as the interviewer was able to ‘poke’ at particular questions and scenarios, for example, how P1 always complained about the games during the intervention (see below). However, it was also a challenge. To minimise the risk of social-desirability, the interviewer often repeated that critical feedback is helpful, and that ‘we have a shared goal, to make better interventions for acalculia, so please tell me how it could be better, or what you would do differently’.

### 2.5. Materials

Material for the sessions were designed to be consistent with the principles of embodiment, playfulness, and ESE. To facilitate the concept of embodiment, all sessions were ‘active’ (there was little ‘sitting’ or work with ‘pen and paper’), and as much as possible, activities were designed to be congruent with the cognitive concepts in the session. This is often inherent in tasks that require physical engagement (e.g., measuring/weighing items, walking distances) and as such easy to include in the design. The principle of playfulness was facilitated by designing the sessions around ‘challenges’ that required a degree of ‘getting things wrong’ before being able to ‘get it right’ (see water-colour challenge), demonstrating that there is more than one correct solution to the problem (see clock reading task), or at least, having an element of luck (very much in contrast to the idea of ‘errorless training’ [68], which has been used several times within acalculia rehabilitation studies [5]).

Lastly, group work was facilitated in several ways. If a task was set for the whole group (e.g., shopping in the market), the group had to work as a team to solve it, but even if tasks were set for individuals (and at times, tailored for individual level, as described in sessions 5 and 6), the focus was not on competition, but rather on group help and support. As such, participants were encouraged to cheer for the success of others (e.g., in the robot driving or cloths fitting tasks) and to seek help from and support each other when they found the task hard (e.g., measuring cooking ingredients or mixing coloured water tubes). The sessions, and tasks within them, are described in detail in Appendix A.

### 2.6. Approach to Analysis

Given the small number of participants, results were not examined for statistical significance, but rather visual analysis was conducted using plots of the acalculia assessment scores. For qualitative analysis, the interviews were video-recorded and transcribed automatically by MS Teams. Following the interview, transcripts were manually checked and corrected by the first author, and gestures from the videos were added to the transcript where appropriate. The resulting transcripts were offered to participants to review, as a form of member checking [69], but neither wished to review their transcripts. Data were then analysed by the first author, supported by the 6th author. Given the small number of participants, we used content analysis [70] with a directed approach [71], to reflect on relevant aspects of the intervention, and consider these alongside the quantitative measures. More specifically, each transcript was coded using NVivo14 Qualitative Data Analysis Software (Lumivero, version 14.24.3, 2024). Codes were then grouped into categories using a mixed inductive and deductive approach, based on aspects of the intervention (what participants remembered/valued/did not like), their perceived outcomes or impact of the intervention, and any theoretical aspects that led the inclusion of the ‘active’ components in the intervention (e.g., playfulness).

## 3. Results

Apart from P4, who did not attend the last three sessions due to an extended summer vacation, all participants attended all sessions, and no adverse events or challenges were noted. That said, session 9, which involved the use of a high-tech simulated environment, was the least successful session. The room got too hot, and participants found it challenging to engage in these conditions. See more details in Table A1.

**Figure 1:**
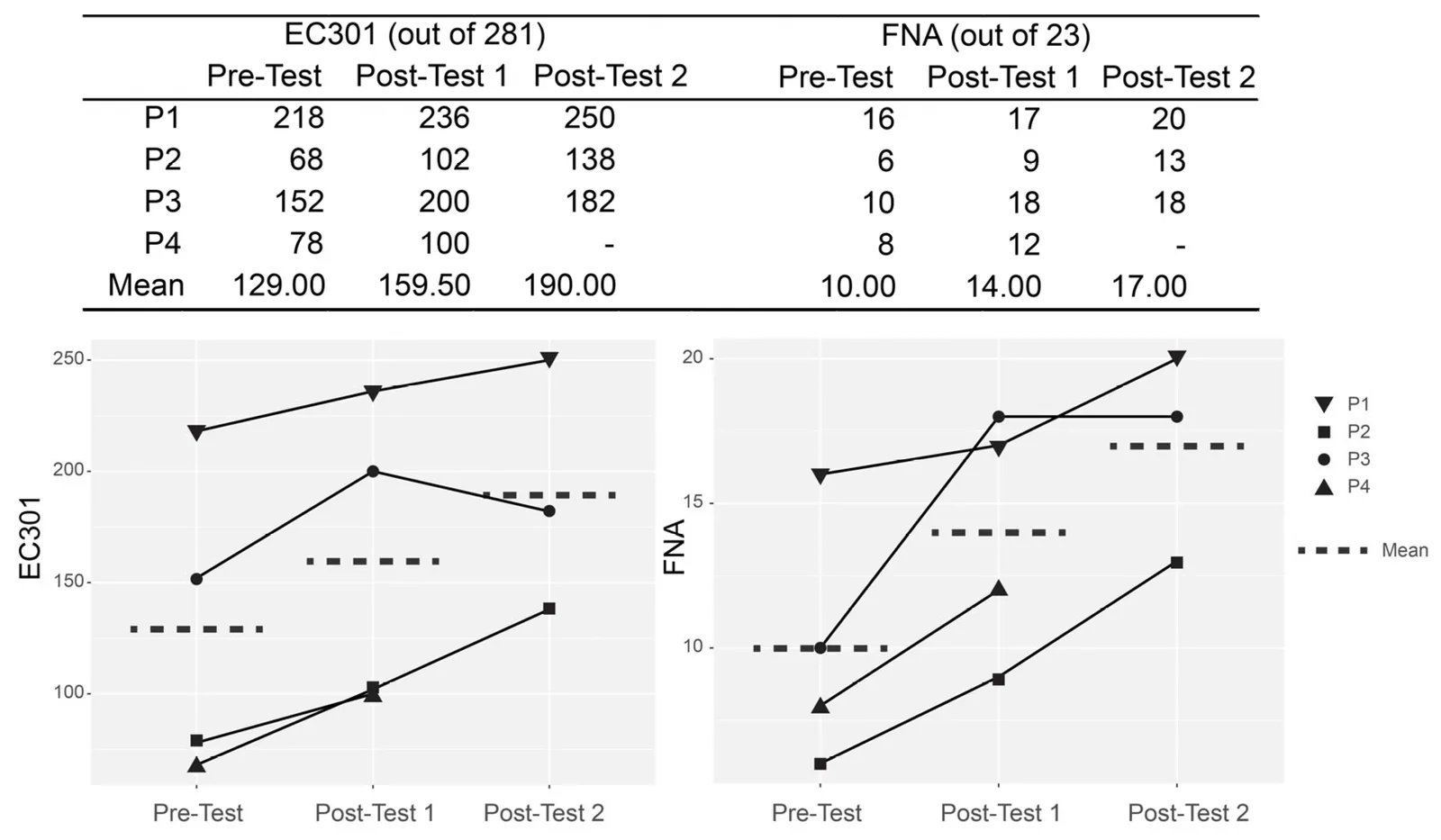
Participants’ scores on the Functional Numeracy Assessment (FNA) and the EC301 battery at baseline (pre-test), after 6 weeks of intervention (post-test 1) and after additional 3 weeks (post-test 2). Group means are shown in dotted lines, and individual scores in solid lines. Note that means for Post-test 2 are based on *n* = 3, while others on *n* = 4. Although there are no normed scores for English speakers on the EC301, all scores fall below the norm for adults with more than 12 years of education, as tested by French, German and Italian speakers. See full scores and more details in Table A3 and Table A4.

Figure 1 illustrates the progress made by each of the participants from baseline (pre-test), to following 6 weeks of intervention (post-test 1) and then following an additional 3 weeks (post-test 2). The scores represent both theoretical number knowledge (as measured by the EC301) and functional number-use (FNA). Reassuringly, both tests show that participants improved over the course of the intervention, except for P3 who scored somewhat lower on the EC301 at post-test 2 compared to post-test 1. However, the score at post-test 2 is still substantially higher than his baseline score, and some variations in performance are expected.

Given the small number of participants, it was not possible to check for statistically significant improvements; however, it is clear that improvements were made and in the case of P2, both EC301 and FNA scores had more than doubled. Importantly, improvements show far-transfer to untrained skills (see Table A3 and Table A4 in Appendix B for specific item scoring).

### 3.1. Qualitative Findings

Findings from the first part of the interviews (the early days post-stroke and the experience of their number deficits and support received) were consistent with our findings in Benn et al. [1]: neither of the two participants were tested for acalculia, nor have they previously received support for their difficulties with numbers, despite P1 asking for it. Also, neither of the participants realised they had difficulties with numbers until later on (P1 realised it approximately 6 months after the stroke, when she was asked by the speech and language therapist about her dress size, and she could not produce the right number. P2 could not recall the moment he found out).

### 3.2. Perception of the Group Intervention Aspect

Both patients reported that all the interventions (e.g., physiotherapy, speech and language, occupational therapy etc.) they had received before, from all professionals, were done on a one-to-one basis (no group interventions), and they both suggested that the group work was useful and that they would have been happy to receive other interventions in this way. P2 suggested that he struggled with some aspects of the tasks the group did, and when asked if he would have preferred the struggles in the group or in one-to-one, he replied “*I think probably as a group. As a group, Better, yeah*.” When asked why, he replied:

“*Help putting ideas.… I think on the basis of, if I’m doing it wrong, you don’t look so bad.*”

P1 suggested that perhaps people with worse aphasia found it harder in the group. However, when the researchers informally asked P3 (whose language was too impaired for him to participate in a full interview) about this, he suggested that in the group, he could see that he had the most significant difficulties using language, but was sometimes better at other things (he was, for example, the best at calculation), and he also enjoyed the interactions. Overall, during the sessions, we did not observe specific issues that related to differences in language skills.

### 3.3. Perspectives on Play and Games

Both participants were positive about the games. This was particularly interesting in the case of P1, who regularly complained throughout the sessions about the games, and demanded every week that we work with ‘pen and paper’. It took weekly persuasions to get her involved in the activities and games. However, in the interview, when asked “*what did you like about the number work that we did?*” she replied: “*Dice, snake and ladders, Bees (gestures of moving to indicate mini-robots’ movement), I want childhood (laughs)*”. When the researcher pointed out that this was something she really did not like during the sessions, and asked what had changed, P1 replied:

“*I think numbers (thinking…gestures of processing in the head by turning the finger near the temple) numbers stayed in. They stayed in because of the games. Yeah, they fitted in—brain (tapping with fingers on the temple)*”.

She then added: “*nine weeks of programme is better for me. For five years—nothing (at this point she makes gesture with hands to show how much more progress in nine weeks compared to 5 years before)*”.

### 3.4. Practical Improvements

Both participants pointed out that outcomes extended beyond the intervention, and that they keep practicing skills in everyday life. P1 suggested that her improved numerical processing encouraged her to handle money. When asked if she now manages the family finance, she laughed and replied: “*No-Shops, restaurants, kids’ money*” (referring to pocket money, and money the children need at school etc.)—things she would not do before. P1 also indicated that she plays more and is more proactive with her children. For example, despite never being a cook (even before the stroke), since the intervention, she had been baking with the kids (fairy cakes), and it helped her make new connections with them. P2 also reported more confidence trying things, as well as having the motivation to continue practicing number skills every day.

### 3.5. Acceptability

The TFA [64] considers seven constructs for evaluating the acceptability of an intervention: affective attitude, burden, perceived effectiveness, ethicality, intervention coherence, opportunity costs, and self-efficacy. Based on the qualitative feedback, we conclude that the intervention was associated with positive affective attitude, the right level of burden (not too much and not too little effort is required), high perceived effectiveness (including P1 changing her views on the effectiveness of the content of the intervention) and improved self-efficacy in both patients.

The qualitative feedback suggests that ethicality, defined as the fit between the individual’s value system and the intervention, could be improved. P1 reported that she perceived that perhaps those with worse aphasia had struggled with the work, which she considered to be problematic, in other words—not in line with her values. Following this up with P3, who had the most severe aphasia symptoms in the group, suggested that this was not the case. To improve acceptability, this should be considered in the future when considering the make-up of group-level interventions, and future group interventions with mixed level of aphasia should examine and report on this aspect, as we found no evidence of such reporting in the literature.

Intervention coherence is defined as the individual’s understanding of the ‘fit’ between the components of the intervention and the intended aim of the intervention. The qualitative feedback suggests that P1 could only retrospectively see how components (i.e., games) were linked to the outcome, and so it is possible that this link could be strengthened in future implementations.

## 4. Discussion

The current feasibility study explored, for the first time, the impact of a 9-week group-intervention for acalculia that is based on the principles of embodied cognition, playfulness and an Enriched Social Environment (ESE)—rather than on traditional drill methodology. All four participants showed improvement in both theoretical and functional number use, extending to untrained skills. Qualitative interviews with two of the participants indicated that they particularly valued the group-aspect of the intervention, and that the use of games and play activity not only contributed to their improved numerical skills, but also to their willingness to engage with further novel tasks in their everyday life, and as such, resulted in improvements in their confidence, and relationships with those around them, contributing to self-reported improvement in overall wellbeing.

Despite considerable progress in the theoretical understanding of how numerical information is learned and processed in the brain (e.g., [72]), there has been little translation of these insights into clinical practice. The participants in this study and those in our previous work [1] had not been assessed previously for numerical difficulties, and therefore became aware of their challenges many months after the stroke/brain-injury (sometimes, after they returned to work, only to then realise they could not perform it [1]). Participants also reported not receiving any support for their numerical deficit, even when they asked for it. This is partly due to the lack of tested interventions for acalculia. Just 16 published studies, with a total of *n* = 31 patients were identified in a recent systematic review [5], with the majority of these studies focusing on re-teaching multiplication facts. Importantly, none of the published work reported using group-work or games as a mode of intervention.

Although the number of participants in the current intervention was small, it is notable that participants improved on a range of theoretical and functional numerical skills beyond aspects they practiced during the sessions. This is termed ‘far-transfer’ and is not commonly observed in stroke rehabilitation [73]. This is also the case for numerical interventions to date, some of which reported very limited transfer of skills from training to testing, even when those skills are closely related. For example, some studies did not observe transfer from training multiplication to improving division (e.g., [8]) or reported that training modality may impact performance (e.g., [13,15]).

We propose that one explanation for the improvement of diverse theoretical and functional skills, often not trained directly during the intervention, is due to the nature of the intervention, which targeted the ‘number sense’ [74] that underpins numerical skills, rather than any one specific number skill. The number sense was targeted by employing embodied and multi-sensory activities where possible, emulating child-like learning, such as finger counting [25,75] and more complex mechanisms that are less well understood, but link Fine Motor Skills (FMS) and Gross Motor Skills (GMS) to mathematical outcomes [27,29,30,31,32,33,34,76,77]. To this end, the activities in the sessions, where possible, included craft and arts, which combine movement (both FMS and GMS), cognition, problem-solving and emotions. These have been argued to further cultivate mathematical thinking by heightening multisensory sensitivities to quantitative thinking through aspects such as engagement with volume, weight, proportion, and symmetry [78]. It has indeed been suggested that sensory-motor activations converge into the Intraparietal sulcus (IPS), to create an abstract number representation [79], and that social-interaction can support this process of abstraction [80].

There is some evidence that settings where cognitive and motor tasks are combined, functional connectivity is increased [81], and it has been suggested that purely intense cognitive training can result in neuroplastic changes in motor areas, due to potential saturation effect [82]. We therefore speculate that the mechanism behind the developmental and the rehabilitation findings that link motor and cognitive improvements may be linked to the neural architecture of the brain, and propose here a testable hypothesis, that (re)learning that involves motor and cognitive processing will result in greater increases in white matter connections, and perhaps stronger IPS activation during numerical tasks. This hypothesis can be easily tested by collecting neuroimaging data before and after interventions that are equivalent in content, but are delivered in either traditional settings or through group-embodied settings. We predict that the latter will result in greater increases in white matter connections, and perhaps stronger IPS activation during numerical tasks.

The impact of the combined cognitive and motor intervention can be explored here through the qualitative comment from P1, which suggested that the numbers simply ‘fitted in’ her brain. This indicates the implicit nature of the learning that took place, as well as supporting the intervention coherence aspect as defined by the Theoretical Framework of Acceptability (TFA; [64]). Indeed, the ‘number sense’, may be impossible to teach through explicit ‘pen and paper’ based tasks. As such, the fact that P1 was able to link between the components of the intervention and the outcomes, represents a great strength of the promise, feasibility and acceptability of the method.

In particular, given that stroke and other types of brain-injury often affect both cognition and movement, more effort should be spent in targeting these simultaneously, by including multi-modality and multi-sensory experiences. Doing this is likely to make rehabilitation more functional, cover currently under-addressed skills (such as numerical-skills), and possibly support more rehabilitation at a more affordable cost. Indeed, within the discipline of speech-language pathology, it has been recognised that multimodality in the way of presenting written and spoken word simultaneously improves naming [83], much like playful group interventions [60]. The current intervention has demonstrated that it is possible to combine multi-sensory and multi-modality work in number training, in group settings, and that this is likely to improve engagement and outcomes.

Qualitative feedback from participants suggested they experienced improvement in overall wellbeing, which may have been achieved through several aspects of the intervention. ESE has been found to have a positive impact on social and wellbeing among brain-injury survivors [58], to benefit cognitive rehabilitation [59,60], and to be more effective for rehabilitation than traditional post-stroke settings [63]. As reported by one of the participants interviewed here, group work also reduces the need for perfect performance, and as such, may contribute to diffused anxiety. This may be particularly important when activities are related to numbers, which are commonly associated with ‘math-anxiety’ i.e., feelings of tension and anxiety arising from the manipulation of numbers and solving mathematical problems [84]. Indeed, we did not measure this in the study, but future work should consider this.

Furthermore, the qualitative feedback suggested that the process of learning from errors as afforded by the concept of playfulness, may have encouraged participants to seek challenges in their daily life, hence increasing learning outside of the intervention sessions. The two participants reported extending the range of activities they have been engaging with, with P1 particularly reporting how this has not only increased her confidence and independence, but also improved her relationships with her children, and as such, her wellbeing and quality of life. Mathematical play lends itself particularly easily to playfulness and risk taking, as it involves playing with possibilities and constraints as offered by materials, tools, negotiation with others, figuring rules, and inhabiting imaginative as-if worlds [56,57]. As playfulness has been shown to reduce stress and increase wellbeing [51,52,53] and it can encourage creativity and seeking out complex and novel challenges within one’s limitations, hence enhancing learning [55], it is imperative that future interventions for acalculia (and perhaps for other deficits) consider embedding this aspect.

### Strengths and Limitations

The current feasibility study has several strengths. It describes a novel approach to rehabilitation, that combines well known principles from cognitive science and education, namely, embodied learning, ESE and games and playfulness, to improve symptoms of acalculia in a diverse group of stroke survivors. Despite the strong theoretical background and the promising outcomes, the work presents several limitations that should be addressed in future work. First, being a feasibility intervention study, the work only involved four stroke-survivors, with full data obtained for three only. While a small sample, group interventions in post-stroke settings are generally rare, and when they do take place, group sizes of four are common (e.g., [59]). In addition, the four participants taking part in this intervention varied in their gender, time post onset, age and severity and type of aphasia, and yet, they all showed improvements on both functional and theoretical tests. However, it should be noted that all were professional, some with high-numeracy level, prior to their stroke (e.g., engineer, biology lecturer), and generalisability to populations with lower baseline numeracy or education should be established. An additional important limitation of the work is the lack of ability to utilise statistical tests to measure improvement, due to the minimal measurements taken. In particular, the lack of multiple baselines (due to the limited availability of assessments and the need to avoid test-learning), makes it difficult to conclude that any changes in performance are anything more than ‘placebo’ or practice effects, or indeed due to spontaneous recovery of linguistic or other skills, which is still possible at 18 months post onset (the EC301 includes some questions that may be influenced by aphasic symptoms, such as verbal repetition of numbers). Indeed, it is unlikely that changes in numerical skills can be acquired as ‘placebo’, and the qualitative feedback explicitly attributed the improvement in numerical skills to the tasks during the intervention, but future work should more carefully control for changes in associated cognition, including language. Furthermore, the sessions were only loosely linked to the tests’ items (e.g., participants never learned how to read football tables, count backwards, or choose the ‘better value’ product), reducing the likelihood of ‘practice effect’. However, one could argue that individual support provided in the final three sessions was more closely linked to test items. The definition of what counts as ‘far transfer’ may be loosely defined. In previous work, transfer of exact and related tasks has been very tight [5]. For example, in multiplication problems, if training an operation, such as 3 × 7, performance was measured on 3 × 7 (exact training) 7 × 3 (close transfer) and 21/3 or 21/7 as far transfer. This has been the standard used by many interventions that consider ‘transfer’ between trained and untrained skills, as they largely used intense repetition of specific skills. Under these definitions, one may consider all the assessments we have used to represent ‘far transfer’ from training, as we have never used exact problems from training within testing, and there was never extensive repetition practice of any one problem. Still, future work should employ a Single Case Experimental Design (SCED, [22,59,85]), to carefully examine progress in small groups, and more carefully define and measure transfer effect on near and far tasks.

Importantly, the design of the current intervention did not enable us to distinguish between the relative contribution of each of the ‘active components’ used (e.g., group, embodied, play), which can be seen as a weakness. Providing three one-to-one sessions for participants further muddles the water. However, the strength of combining these elements is in the enjoyment of the tasks, and the practicalities of the delivery: none of the sessions were delivered by clinicians, which suggests that this intervention could be delivered in community settings, greatly improving access and reducing cost of interventions, hence improving rehabilitation prospects. We suggest that combining group-setting with home self-practice based on individual needs and goals would provide ample opportunities for improvements without increasing delivery cost. Lastly, participants differed in their language and possibly other skills, but we did not measure most of these at baseline, nor did we measure wider outcomes, such as language, executive function, spatial processing, wellbeing or physical changes. Future work should examine this, and whether there are costs (e.g., challenges in group-interactions due to communication differences) and/or ‘added value’ to wider cognitive, physiological and/or wellbeing measures by combining methods compared to using these separately (i.e., more consideration to multi/transdisciplinary work).

## 5. Conclusions

This is the first study to examine the impact of an acalculia intervention that is not based on individual work or primarily focused on developing specific skills through intense repetition. Instead, we present here the first ever intervention for acalculia that is group-based, embodied, multi-sensory and playful. Importantly, beyond being fun and effective for improving both functional and theoretical numerical concepts with evidence for far-transfer, the qualitative feedback from participants suggests that the methods used were acceptable, enjoyable, and had encouraged exploration and learning outside of the sessions. This likely contributed to participants’ confidence, independence, and relationships with those around them, resulting in overall improvement in wellbeing. Despite several limitations, this feasibility study demonstrates that group interventions that are based on solid current theoretical frameworks could provide a cost-effective way to address multiple challenges that are common after a stroke or a brain-injury, including cognition and wellbeing. Lastly, we propose a testable hypothesis suggesting that intersecting multiple sensory-motor neural pathways simultaneously, is likely to facilitate neuroplasticity, which may be observed through increased white-matter connectivity, and contribute to the establishment of abstract representation of ‘number sense’.

## Figures and Tables

**Table 1:** Stroke Survivors Participant Information.

Participant ID	Age	Gender	TPO * (Months)	Aphasia	Occupation (Pre-Brain-Injury)	Physical Disability
P1	46	F	62	Speech apraxia	Nurse	Hemiplegia affecting the non-dominant hand, unstable walking
P2	73	M	20	Fluent Wernicke’s aphasia	Engineer	None
P3	50	M	18	Global aphasia	Cyber security consultant	Hemiplegia affecting the dominant hand, and walking aided by stick
P4	77	M	18	Mild to moderate expressive and receptive aphasia.	Biology lecturer	None

* TPO: Time post onset.

## Data Availability

The data presented in this study are available on request from the corresponding author due to ethical constraints.

## Abbreviations

The following abbreviations are used in this manuscript:

ESE	Enriched Social Environment
FMS	Fine Motor Skills
FNA	Functional Numeracy Assessment
GMS	Gross Motor Skills
RCT	Randomised Control Trials
SD	Standard Deviation
TFA	Theoretical Framework of Acceptability
TPO	Time Post Onset
WAB	Western Aphasia Battery

## Appendix A

**Table A1:** Materials and procedures used in each of the nine sessions.

Session Number and Focus	Tasks Used	Relevant Image
1. Personal introduction: age, address, telephone-number etc.	The session started with the group sitting in a circle, and using a large foam dice for an introduction game, where each individual in turn rolled the dice and depending on the number, they introduced an aspect of themselves—e.g., if rolling the number 2, they may say: “I have two brothers”. The number used could be for any numerical information—dates, telephone numbers, addresses, etc. As there was no table, participants had to bend to lift the large dice. The dice-size was used to encourage the use of two hands, though this was not always possible, and the foam material made it possible to hold in one hand if preferred. The task took almost the whole session, participants struggled to think of the information, and some struggled saying it (due to aphasia). It encouraged creative communication skills.	
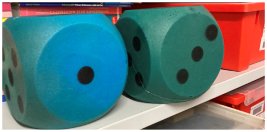
2. Counting and number-line	Using a floor mat, little play robots (bees) and a large dice, participants played a ‘bug race’. The game involved throwing the dice and progressing on the board the number of steps as indicated by the dice. Moving on the board was done by ‘programming’ the robot bee, by pressing a button to indicate the number of steps forward and any turns that may be needed. Each player had their own bug, and the first bug to reach 100 (or beyond) was the ‘winner’. A supportive atmosphere, of cheering when the bee arrived at the right destination made the activity a group, rather than competitive activity.	The large mat was designed with several implicit messages: All the 10s were bold. Odd and even numbers were in different shades The ‘teens’ were circled to indicate their ‘special’ status linguistically The colours got darker as numbers got bigger, in line with literature suggesting this is a common association [86].
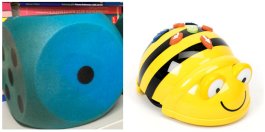
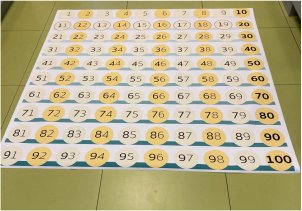
3. Number production (transcoding)	Three felt dart boards were prepared with one board having figures in the 100 s (100 in the outer layer 400 in the centre) one in the 10s (10–40) and one in the single (1–4). Participants had to throw five balls at each board, write their scores on a sheet (in the centre), and then make up five full numbers based on their throw (e.g., 400 + 30 + 1 = 431). Participants then practiced, in a group, ‘reporting’ the numbers they produced.	
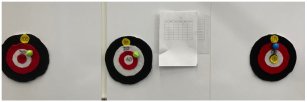
4. Reading and telling the time	Using plastic clocks where the hands only moved clockwise, participants were sat in a circle and in turn, took a note out of an envelope. The note included a time, specified in different formats (see examples on the right). Having pulled a time from the envelope, the participant had to read it aloud, and everyone had to set their clocks to that time. Everyone then, in turn, had to say the sentence ‘the time is XXX’ but in a way that was different to the person before them. For example, using ‘15 min past’ or ‘quarter past’ or ‘45 min to’ or using a 24 h clock, etc. This was done as a team challenge—find another way to say the same time.	
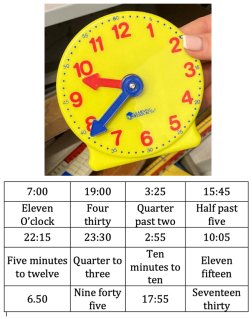
5. Number naming and recognition	The session started with placing discs on a number line (in pairs), and then continued into a variation of a bingo game. Participants received a unique grid (3 × 3) with numbers and simple calculations (the difficulty of numbers and calculation slightly varied by individual ability, according to baseline FNA and EC301 scores). They were given some time to solve the calculations (with or without help, as needed) and write their answers on their grid. An envelope with only the numbers on the boards (such that every number retrieved was a ‘hit’) was passed around, and in turn, each participant had to retrieve a note and read it aloud. Most participants struggled, and the team worked together to articulate the numbers. Each individual then had to identify it on their board and mark it off (with help if needed). The first full board got a clap.	
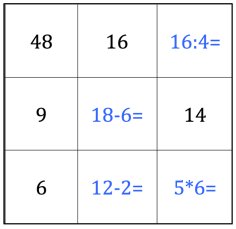
6. Executive function and calculation	Each participant received a recipe. All the recipes were for the same chocolate and biscuit ‘balls’, but the difficulty of the task varied slightly by ability (based on baseline test)—while some received the exact recipe, others received a recipe that had to be ‘doubled’. Participants had to measure the amounts (using scales) and follow the recipe (very simple—just mix all the ingredients and form into equal-size balls). The resultant food was used for an ‘end of programme’ celebration.	
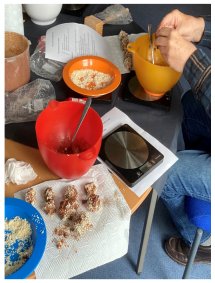
4 weeks break during which testing took place		
7. Estimation: quantity, greater/smaller than	Coloured water containers were pre-set on the table prior to participants arriving at the session (top image). After a short discussion of colour mixing (what happens if we mix red and yellow in equal amount? What happens if we then add? Etc), each participant was given a cup with their own unique colour. Using tubes to measure and mix coloured water, participants had to create a ‘recipe’ for their colour, which was then ‘tested’ by the team. Participants often worked in pairs, or with support, to compensate for mobility difficulties.	
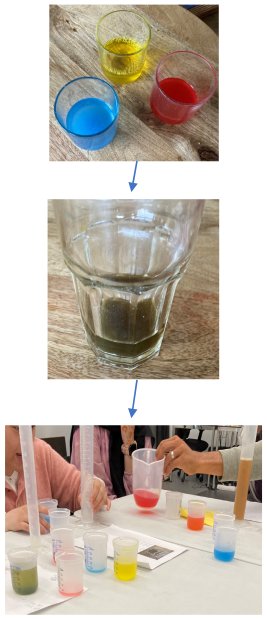
8. Measuring and visuo-spatial processing	Cardboard human figures were hung on the wall prior to the start of the session, and the room was set up with tables with fabrics, scissors, and measuring devices (measuring-tapes, rulers etc). The task was to measure the cardboard figure, draw the required shapes on the fabrics, and ‘make clothes’ for the figures.	
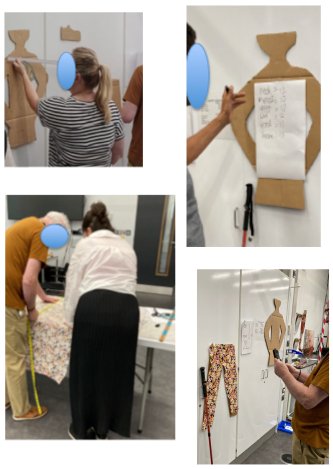
9. Immersive market environment	The group received a ‘shopping list’ and ‘money’ and they had to go through an immersive environment representing a market. Interaction with the environment took place by touching the walls—either typing on a giant keyboard or pressing ‘buttons’. While fun, this session was perhaps the least successful. The task was too difficult, and the room got very hot. Low-tech solutions were more effective.	
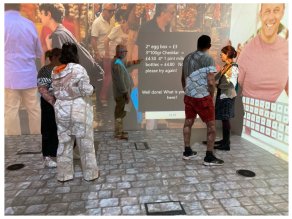

## Appendix B

**Table A2:** Western Aphasia Battery Scores for each participant.

Participant	WAB-AQ	WAB-LQ	WAB-CQ
P1	88.8	90.3	91.4
P2	66	53.3	59.9
P3	50.2	52	60.1
P4	65.6	63.2	67.7

NOTE: WAB: Western Aphasia Battery, AQ: Aphasia Quotient, LQ: Language Quotient, CQ: Cognitive Quotient.

**Table A3:** Functional Numeracy Assessment (FNA) scores per participant at T0, T1 and T2.

FNA			T0				T1				T2		
Question	Description	Max Score	P1	P2	P3	P4	P1	P2	P3	P4	P1	P2	P3
1	What time is it?	1	1	0	0	0	1	0	1	1	1	1	1
2	How much battery is left on the clock?	1	1	0	0	1	1	1	1	0	1	0	1
3	How many draws were there this week?	1	1	0	0	1	0	0	1	1	1	1	1
4	How many teams did not score in their matches?	1	1	0	0	0	1	1	0	0	1	0	0
5	How many points does the third team have?	1	0	1	0	0	1	1	1	0	1	0	0
6	How many wins does Arsenal have?	1	1	0	0	0	1	0	0	0	1	1	0
7	Can you tell me Garry’s phone number?	1	0	0	0	0	1	0	1	1	1	0	1
8	Whose number ends with “176”?	1	0	1	0	1	1	1	1	1	1	1	1
9	Here is Jane’s phone number, can you type it below?	1	1	1	1	0	1	0	1	0	1	1	1
10	You have 720 g of flour. How many scones can you bake?	1	0	0	0	0	0	0	0	0	0	0	0
11	Which orange juice is the cheapest one?	1	1	1	0	1	1	1	1	1	1	1	1
12	You have £60. You want 4 bottles of perfume. Which 4 can you get?	1	1	0	0	0	0	1	1	1	1	1	1
13	Which blueberries are better value?	1	1	1	0	1	0	1	1	0	1	1	1
14	Here is a promotion. You buy 3 items and get the cheapest one for free. Which one of these items do you get for free?	1	1	0	0	0	1	0	0	1	1	1	0
15	You are buying these two items. How much will you pay?	1	1	0	1	0	1	1	1	1	1	1	1
16	Your shopping costs £4.20. Which box has enough money?	1	0	0	1	1	1	0	1	1	1	1	1
17	You bought a book and it cost £12.49. You paid with a £20 note. How much will you receive as change?	1	1	0	1	0	0	0	1	0	0	0	1
18	You catch the train at Finsbury Park. You travel seven stations towards Brixton. Where do you get off?	1	1	0	1	0	1	0	1	1	1	0	1
19	You are at Brixton and need to go to Warren Street. How many stations do you have to travel?	1	1	0	1	0	1	0	1	0	1	0	1
20	Can you identify the correct wifi password?	1	1	1	1	1	1	0	1	1	1	1	1
21	It’s 3:25 pm now, the train to Basingstoke leaves at 16:12, how many minutes do you have before the departure?	1	0	0	1	0	1	0	1	0	1	0	1
22	You are making risotto. You have 5 guests. How much risotto rice do you need?	1	0	0	1	0	0	0	0	0	0	0	1
23	Which city had the highest recorded temperature in February?	1	1	0	1	1	1	1	1	1	1	1	1
	Total row score:	23	16	6	10	8	17	9	18	12	20	13	18
	% accurate		69.57	26.09	43.48	34.78	73.91	39.13	78.26	52.17	86.96	56.52	78.26

**Table A4:** EC301 scores per participant at T0, T1 and T2 (% accuracy).

EC301		T0				T1				T2		
Question	Description	P1	P2	P3	P4	P1	P2	P3	P4	P1	P2	P3
1	Spoken verbal counting	50	25	25	25	100	50	75	75	100	50	75
2 *	Arabic digit counting	100	0	0	100	100	0	100	100	100	100	100
3 *	Written verbal counting	100	50	50	100	100	100	100	100	100	100	100
4 *	ED Small sets (6, 4, 5) on Dominoes	100	100	100	66.67	100	100	100	100	100	100	100
5 *	ED Small sets (4, 6, 5) on random spatial arrangements	100	66.67	100	100	100	100	100	100	100	100	100
6	ED Medium size sets (11, 8, 10) on segmentable arrangements	100	100	100	66.67	100	100	100	100	100	100	100
7	ED Medium size sets (10, 8, 11) on random arrangements	100	66.67	100	100	100	100	100	100	100	100	100
8	ED Medium size sets (9, 7, 12) on linear arrangements	100	100	100	100	100	100	100	100	100	100	100
9 *	NT Oral repetition of numbers	83.33	0	16.67	16.67	83.33	16.67	16.67	0	83.33	33.33	33.33
10	NT from Arabic digit to written verbal numbers	33.33	0	0	0	66.67	0	0	0	66.67	0	33.33
11 *	NT Reading aloud numbers in Arabic digit forms	50	0	16.67	0	50	16.67	66.67	16.67	83.33	33.33	66.67
12	NT Writing to dictation written verbal numbers	16.67	0	0	0	50	0	16.67	0	33.33	0	16.67
13 *	NT Reading aloud numbers in written verbal forms	83.33	0	33.33	16.67	100	16.67	66.67	0	100	16.67	33.33
14	NT Writing to dictation Arabic digit numbers	66.67	0	83.33	0	66.67	0	83.33	0	83.33	0	66.67
15	NT From written verbal to Arabic digit numbers	83.33	0	100	0	83.33	0	100	0	100	0	83.33
16 *	Arithmetical Signs (=, ×, +, −) Naming	100	50	25	75	100	75	75	50	100	75	100
17 *	Arithmetical Signs (=, ×, +, −) writing from dictation	100	75	75	75	100	100	100	50	100	100	100
18	Magnitude Comparison of numbers (Arabic digit code)	87.50	37.50	25	37.50	87.50	12.50	100	37.50	87.50	75	100
19	Magnitude Comparison of numbers (Written verbal code)	100	0	87.50	0	87.50	0	75	0	100	37.50	50
20	Mental calculation on spoken verbal numbers	87.50	0	25	0	100	12.50	62.50	0	87.50	50	50
21	Mental calculation on Arabic digit numbers	100	37.50	100	12.50	100	62.50	75	50	87.50	62.50	62.50
22	Approximation of the result of an operation	87.50	0	100	25	100	50	100	62.50	87.50	87.50	100
25	Place multidigit numbers to perform an operation	75	0	0	0	50	0	0	0	100	0	0
26	Written calculation. Additions	50	0	0	0	100	0	100	0	100	0	0
27	Written calculation. Subtractions	50	0	0	0	100	0	0	0	100	0	0
28	Written calculation. Multiplications.	0	0	0	0	28.57	0	0	0	28.57	0	0
29	Magnitude approximations of pictured stimuli	50	33.33	66.67	16.67	33.33	66.67	66.67	33.33	100	66.67	50
30	Contextual magnitude judgments	100	40	100	60	100	40	100	80	100	80	100
31 *	Precise numerical knowledge	100	66.67	66.67	66.67	100	83.33	100	100	100	66.67	83.33

* Indicates that normative data collected from French, German and Italian speakers, indicated that minimum 97% of adults with minimum 12 years of education achieved a 100% accuracy on these items. Also, items 23 (Placing Arabic numerals: 86, 48, 32, 5, 62) and 24 (Placing spoken numbers: 6, 47, 33, 87, 61) on a number line from 1–100 were not tested due to using online testing, and it was not clear if (or how) digital placement could be made equivalent to physical placement. Note: ED: Enumeration of dots; NT: Numerical Transcoding.
